# Ventriculoperitoneal shunt patients and glaucoma: a cohort analysis of the NPH registry

**DOI:** 10.1186/s12987-024-00558-0

**Published:** 2024-07-09

**Authors:** Benjam Kemiläinen, Kai Kaarniranta, Ville Leinonen

**Affiliations:** 1 Neurosurgery of NeuroCenter, Unit of Neurosurgery, Institute of Clinical Medicine, Kuopio University Hospital, University of Eastern Finland, Kuopio, Finland; 2 Department of Ophthalmology, Institute of Clinical Medicine, University of Eastern Finland and Kuopio University Hospital, Kuopio, Finland; 3 Department of Molecular Genetics, Faculty of Biology and Environmental Protection, University of Lodz, Lodz, Poland

**Keywords:** Idiopathic normal pressure hydrocephalus, Glaucoma, Ventriculoperitoneal shunt, Amyloid-β, Hyperphosphorylated tau

## Abstract

**Background:**

Idiopathic Normal Pressure Hydrocephalus (iNPH) is a chronic condition affecting the elderly. It is characterized by a triad of symptoms and radiological findings. Glaucoma is the leading cause of irreversible blindness worldwide. Earlier studies have proposed that the rate of glaucoma is higher in iNPH patients, and of a possible link between ventriculoperitoneal shunt (VP) treatment and the development of glaucoma.

**Objectives:**

This study aimed to determine the prevalence of glaucoma among iNPH patients and assess the impact of VPs on glaucoma prevalence.

**Methods:**

A cohort study was conducted at Kuopio University Hospital (KUH), including 262 patients with a ventriculoperitoneal shunt. Clinical data were obtained from the Kuopio NPH Registry and medical records. Patients were grouped by iNPH status: iNPH (+) – probable/possible iNPH (*n* = 192), and iNPH (-) – other causes of hydrocephalus (congenital, secondary, obstructive) (*n* = 70). We conducted statistical analysis using the Independent Samples T-test, Fisher’s exact test, and Pearson Chi-Square. We compared demographics, glaucoma prevalence, brain biopsies positive for Amyloid-β (Aβ) and hyperphosphorylated tau (HPτ) as well as comorbidities for hypertension and diabetes medication. Age stratification assessed glaucoma prevalence in the full cohort.

**Results:**

Both iNPH (+) and iNPH (-) groups had comparable demographic and comorbidity profiles. The prevalence of glaucoma in the iNPH (+) group was 11.5% (*n* = 22) and 11.4% (*n* = 8) in the iNPH (-) group without a statistically significant difference (*p* = 1.000). Brain biopsies positive for Amyloid-β (Aβ) and hyperphosphorylated tau (HPτ) were similar.

**Conclusions:**

Neither shunted iNPH patients nor those with a comorbid condition other than iNPH showed a markedly higher prevalence of glaucoma. Instead, both groups exhibited age-related increases in glaucoma prevalence, similar to the trends observed in population-based studies. Our data does not suggest a correlation between VP shunts and an elevated rate of glaucoma.

## Introduction

Idiopathic Normal Pressure Hydrocephalus (iNPH) is a chronic disease affecting the elderly population. It is characterized by Hakim’s triad [1], which consists of gait disturbance, cognitive impairment, and urinary incontinence. It is associated with radiologically confirmed ventriculomegaly, periventricular hyperintensities, a disproportionate widening of the sylvian fissure compared to other sulci, and localized sulcal enlargement not attributed to general atrophy [2]. Shunt surgery can alleviate the symptoms of Hakim’s triad [3]. Unlike secondary NPH, currently the specific pathophysiology and pathogenesis of iNPH remains undiscovered, but several mechanisms have been proposed [4]. The prevalence of NPH increases with age. In a recent study, the prevalence among 70-year-olds was 1.5% [5], while among people aged 80 and older, the prevalence rises to 5.9% [6].

Glaucoma is the leading cause of irreversible blindness in the world [7]. It is a conglomerate of progressive optic neuropathies characterized by the gradual degeneration of the retinal nerve fiber layer (RNFL) and retinal ganglion cells followed by characteristic changes in the optic nerve head [8]. Glaucoma classification is primarily categorized into two major types based on anatomical and pathophysiological factors: primary and secondary glaucoma [9]. Both can be further subdivided. Primary glaucoma encompasses open-angle glaucoma with subtypes of primary open-angle glaucoma (POAG) [8] including pseudoexfoliation syndrome (PEX) associated pseudoexfoliative glaucoma (PEXG) [10, 11] and normal tension glaucoma (NTG). Secondary glaucoma is associated with closed-angle conditions in the anterior chamber that lead to elevated intraocular pressure (IOP), often with known etiology. This category includes primary angle-closure glaucoma (PACG) and secondary closed-angle glaucoma.

The global prevalence of glaucoma is estimated to be 3.54%, with POAG accounting for 3.05% and PACG for 0.50%. In Europe, the overall prevalence of glaucoma is estimated at 2.93%, with POAG representing 2.51%, and PACG 0.42% of cases [12]. In Finland there have been few estimates on glaucoma prevalence. In 1995 a population-based study found the prevalence of glaucoma to be 12% among inhabitants aged 70 years or older (*n* = 500) in northern Finland. 8% of that population had been diagnosed with glaucoma before the survey conducted in the study by Hirvelä et al. [13]. The Finnish Institute for Health and Welfare conducted two nationwide health surveys in 2000, followed by a subsequent assessment in 2011. The 2011 summary reported a verified glaucoma prevalence of 2.57% among the study population aged 30 years and older. However, the prevalence of glaucoma showed a substantial rise in correlation with age, exceeding 10% among individuals aged 75 years and older [14]. It has been estimated that about half of glaucoma cases go undiagnosed [15, 16], with POAG being more likely to remain undiagnosed than PEXG [14].

Some of the known risk factors for glaucoma include gender, genetics, family history, smoking, race, systemic hypotension, hypertension, vasospasm, systemic and topical steroids, obstructive sleep apnea syndrome, age and frailty, myopia, migraine, pigmentary dispersion syndrome, pseudoexfoliation syndrome, diabetes, exposure to IOP fluctuation and increased IOP [17]. Obstructive sleep apnea is known to be associated with both glaucoma [18] and iNPH [19]. More recent research suggests that iNPH patients treated with ventriculoperitoneal (VP) shunts may have a higher incidence of normal-tension glaucoma (NTG) [20, 21]. Additionally, patients with iNPH and those with both NTG and iNPH exhibit shallower optic disc cupping compared to NTG patients without iNPH [22].

In this cohort study, our primary objective was to determine the prevalence of glaucoma among iNPH patients. Our secondary goal was to assess the impact of VP shunts on glaucoma prevalence.

## Methods and patients

This cohort study was conducted at Kuopio University Hospital (KUH). The study was approved by The Kuopio University Hospital Research Ethics Committee (276/2016) and was conducted according to the tenets of the Declaration of Helsinki. All participants in this study provided written informed consent before being enrolled in the NPH registry.

KUH is one of the five university-affiliated hospitals in Finland. It operates as an academic, non-profit institution that receives public funding. It serves as a tertiary care center. KUH catchment area includes four central hospitals and has a catchment population of approximately 850,000. Patients with suspected iNPH undergo an initial evaluation by a neurologist. If the patient displays one to three symptoms of Hakim’s triad and has enlarged brain ventricles (Evans index > 0.3) in their MRI or CT imaging, and if no other clearly defined reason for the symptoms is identified, they are referred for further neurosurgical investigation. Neurosurgical investigations for NPH are exclusively conducted at KUH within the aforementioned catchment population.

Kuopio NPH Registry has conducted prospective data collection since March 2008. The registry contains clinical information and long-term follow-up visits. Since 2010, it has included systematic CSF sampling preoperatively and brain biopsy samples during shunt surgery. Brain biopsy procedure and the process of selecting patients for shunting [23] have been described in detail in previous studies. Immunohistochemical analyses of the biopsies were performed by an experienced neuropathologist using light microscopy and the results were graded either as present or absent for cellular or neuronal immunoreactivity for amyloid-β (Aβ) and hyperphosphorylated tau (HPτ). Detailed descriptions of the immunohistochemical analyses have been previously provided [24]. The cut-off, based on electronic patient information system reliability, was set to January 2009. Between January 2009 and June 2023, 644 patients were included in the NPH registry. To obtain current information on glaucoma, 355 patients residing outside the KUH patient information system were excluded. The 27 patients, who did not undergo shunt placement, were subsequently excluded from the study.

**Fig. 1:**
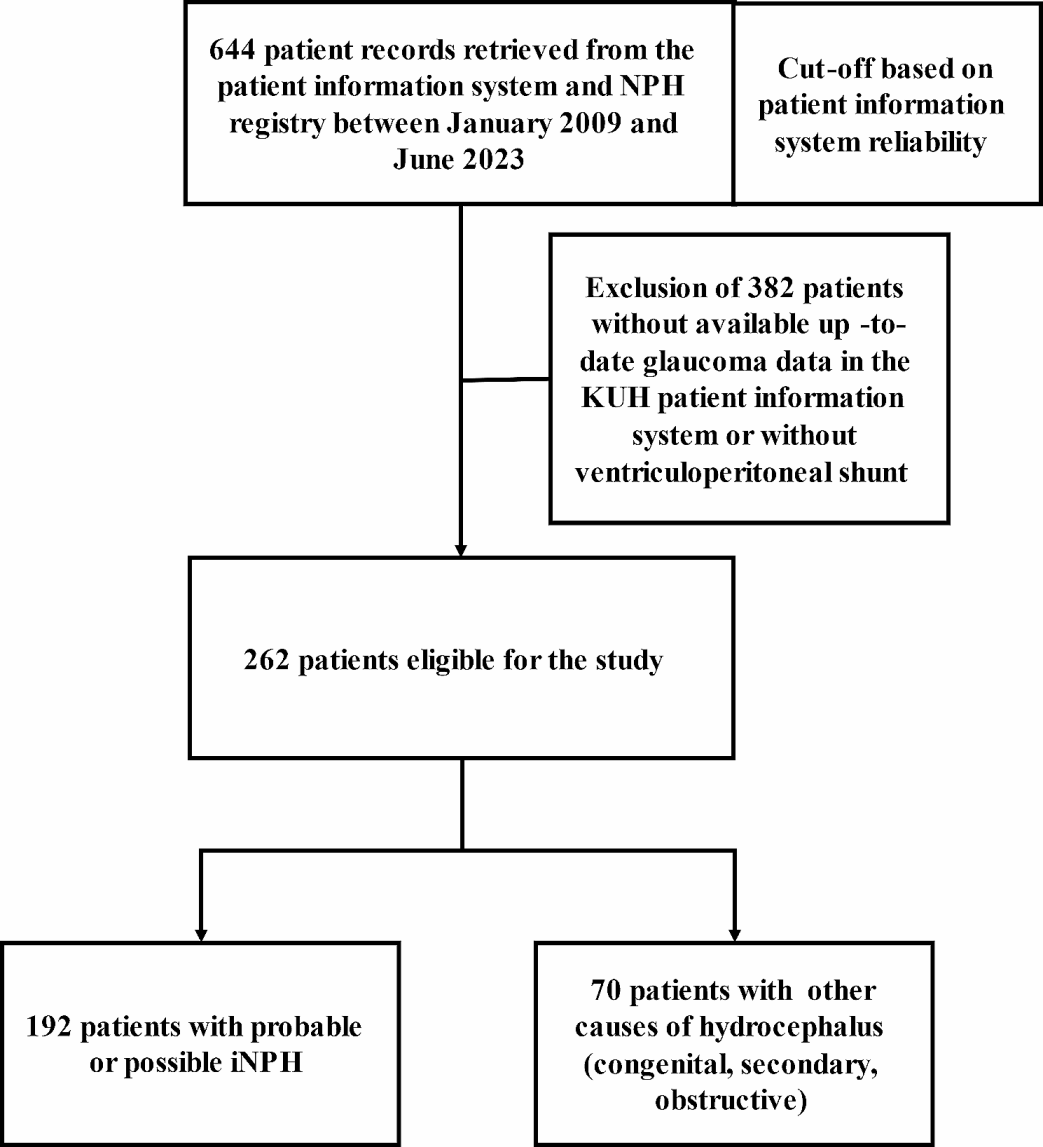
Patient selection flowchart

After exclusion, 262 patients were eligible for the study (Fig. 1.). The full patient cohort demographics prior to grouping, including the age at the time of gathering glaucoma data in September 2023 or age at death, gender, and history of hypertension or type 2 diabetes (T2DM) were obtained. There were no cases of type 1 diabetes among the study population. Data for hypertension and T2DM have been obtained at the time of NPH registry admission. To assess the prevalence and types of glaucoma, we individually reviewed the medical records, the NPH registry, and the electronic prescription system. We identified the history of glaucoma and the use of glaucoma medication. Obtaining precise timing data regarding glaucoma diagnosis and type is challenging due to the decentralized nature of glaucoma diagnosis across both private and public healthcare sectors. Glaucoma was categorized into 6 subtypes comprising POAG, PEXG, NTG, PACG, secondary glaucoma and unspecified glaucoma. The prevalence of glaucoma was stratified by age in the full patient cohort, with age categories including 55–64 years, 65–74 years, and 75 years and older.

Patients were then categorized into two groups based on their iNPH status. The iNPH (+) – probable/possible iNPH group (*n* = 192) was formed by patients whose primary diagnosis indicated possible or probable iNPH. The comparison group, iNPH (-) - other causes of hydrocephalus (congenital, secondary, obstructive), included 70 patients. The probable or possible iNPH diagnosis has been performed using the international diagnostic guidelines based on clinical history, brain imaging, physical findings, and physiological criteria [25]. Comorbid conditions other than iNPH included neurodegenerative diseases prior to hydrocephalus, trauma, intracranial hemorrhage, and hydrocephalus due to obstruction, inflammation, congenital hydrocephalus or malignancy.

### Statistical analysis

The data were analyzed with Statistical Package for Social Sciences (IBM Corp. Released 2020. IBM SPSS Statistics for Windows, Version 27.0. Armonk, NY: IBM Corp). Statistical significance was considered when (*p* < 0.05). To compare variables between the iNPH (+) and iNPH (-) groups, the following statistical tests were employed. The Independent Samples T-test was applied to age, Pearson Chi-Square was used for glaucoma type, and Fisher’s Exact Test was employed for the other non-continuous variables. Additionally, Levene’s test for equality of variances was conducted as part of the Independent Samples T-test to assess differences in variances.

## Results

**Fig. 2:**
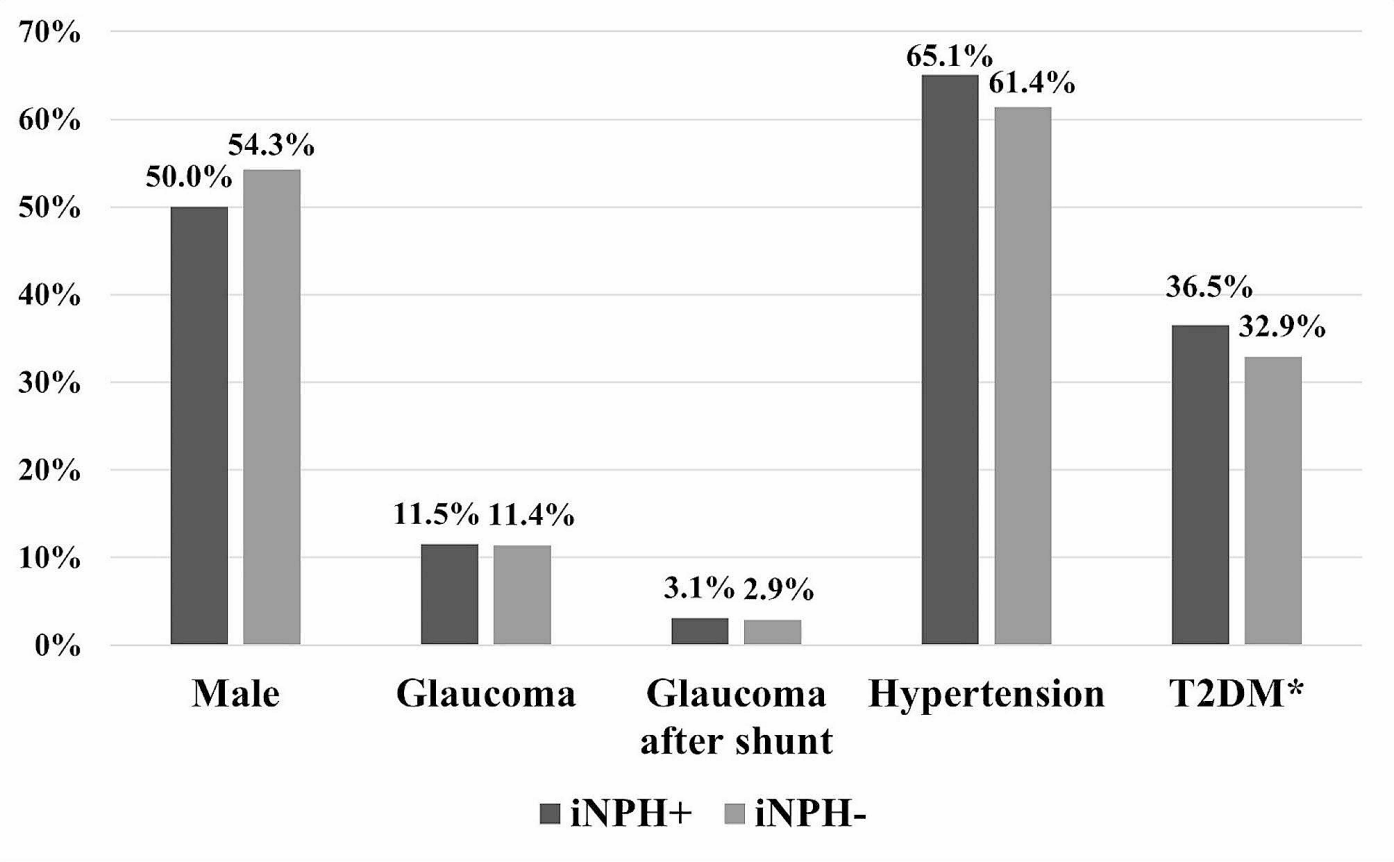
Visualization of gender, glaucoma, and cardiovascular demographics between iNPH (+) and iNPH (-) groups. *Type 2 diabetes. Data missing from T2DM *n* = 2 (1 per group)

Table 1. presents the comparative analysis of group demographics, comorbidities, and glaucoma prevalence between the iNPH (+) and iNPH (-) groups. Figure 2. shows a visualization of the patient demographics. Both groups displayed comparable demographic profiles and variables. The mean age of the iNPH (+) group was 79.4 years and iNPH (-) 78.7 years without a statistically significant difference (*p* = 0.512). Both groups showed comparable representation of male and female participants, with the iNPH (+) group consisting of 96 males (50.0%) and the iNPH (-) group with 38 males (54.3%) without a statistical significance (*p* = 0.578). Comparison of glaucoma did not demonstrate statistically significant differences between the groups (*p* = 1.000). The NPH (+) group had 22 cases of glaucoma (11.5%), whereas the NPH (-) group presented 8 cases of glaucoma (11.4%). The iNPH (+) and iNPH (-) groups demonstrated variations in the distribution of glaucoma types, although no statistically significant difference was identified. In the iNPH (+) group POAG accounted for 40.9% (*n* = 9), followed by PEXG at 27.3% (*n* = 6), NTG at 12.5% (*n* = 1). Additionally, PACG and secondary glaucoma each represented 4.5% (*n* = 1) of cases, while unspecified glaucoma cases accounted for 22.7% (*n* = 5). In contrast, in the iNPH (-) group, POAG comprised 12.5% (*n* = 1) of cases, PEXG was the most prevalent at 75.5% (*n* = 6), and NTG accounted for 12.5% (*n* = 1). There were no cases of PACG, secondary glaucoma or unspecified glaucoma in the iNPH (-) group.

The prevalence of comorbidities was similar between the groups. In the iNPH (+) group, 125 participants (65.1%) had hypertension, and 70 (36.5%) had T2DM, while the iNPH (-) group was comprised of 43 participants (61.4%) with hypertension and 23 (32.9%) with T2DM. Comorbidities did not indicate any statistical significance for hypertension (*p* = 0.663) or T2DM (*p* = 0.662) between the groups. At the time of collecting comorbidity data for hypertension and T2DM, the mean age was 74.6 (range 53–87) for the NPH (+) group and 73.8 (range 40–87) for the NPH (-) group. Brain biopsies for Aβ and HPτ did not display statistically significant differences between the groups (*p* = 0.252, *p* = 0.285, respectively).

**Table 1:** Group comparison

Variables	iNPH (+)		iNPH (-)		Sig. (2-sided)
	*n* (%)		*n* (%)		
Patients	192		70		
Gender (Male)	96(50.0)		38(54.3)		0.578
Glaucoma	22(11.5)		8(11.4)		1.000
Glaucoma after shunt	6(3.1)		2(2.9)		1.000
Glaucoma type*					0.074
POAG	9(40.9)		1(12.5)		
PEXG	6		6(75.5)		
NTG			1(12.5)		
PACG	1(4.5)				
Secondary glaucoma	1(4.5)				
Unspecified glaucoma	5(22.7)				
Hypertension	125(65.1)		43(61.4)		0.663
T2DM **	70(36.5)		23(32.9)		0.662
Aβ positive brain biopsy**	85(47.5)		38(56.7)		0.252
HPτ positive brain biopsy**	33(18.4)		17(25.4)		0.285
Age (years)	**Mean ± SD**	**Range**	**Mean ± SD**	**Range**	**Sig. (2-sided)**
	79.4 ± 7.2	55–97	78.7 ± 7.8	55–92	0.512

*Of glaucoma cases; **Data missing T2DM *n* = 2 (1 per group), **Data missing Aβ positive brain biopsy *n* = 16 and HPτ positive brain biopsy *n* = 16 (13 from iNPH + and 3 from iNPH group). Fisher’s exact test was used for Gender, Glaucoma, Glaucoma after shunt, Hypertension, T2DM, Aβ positive brain biopsy and HPτ positive brain biopsy; Pearson Chi-Square was used for Glaucoma type and Independent Samples T-test was used for age; POAG, primary open angle glaucoma; PEXG pseudoexfoliative glaucoma; NTG, normal tension glaucoma; PACG, primary angle-closure glaucoma; NPH, normal pressure hydrocephalus; T2DM, type 2 diabetes; Aβ, Amyloid-β; HPτ, hyperphosphorylated tau

**Fig. 3:**
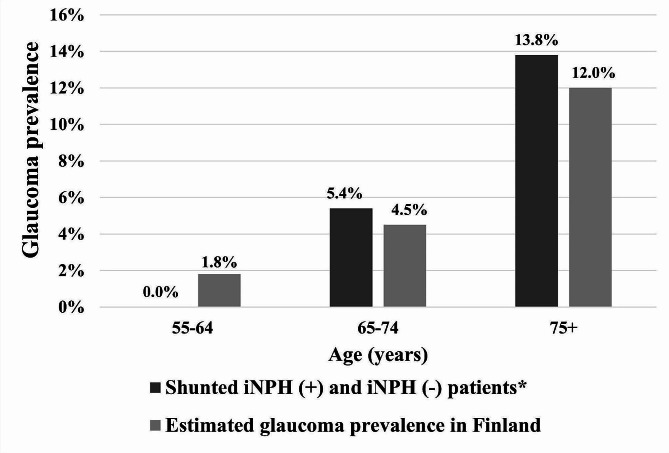
Age-stratified glaucoma prevalence in the study population (*n* = 262) in comparison to presumed glaucoma prevalence in Finland. Modified from Purola et al. 2022 with permission. *n = 10, 56 and 196, respectively

In Fig. 3, the prevalence of glaucoma across the entire study population is shown in an age-stratified manner. Within the 55–64 age category, there were no cases of glaucoma. In the 65–74 age category, the prevalence was 5.4%, increasing to 13.8% in the age category of 75 and older. Estimated glaucoma prevalence for the Finnish population is displayed as a reference. In the full study cohort iNPH constituted the primary diagnosis for 192 patients (73.3%), while 30 individuals (11.5%) had a diagnosis of glaucoma, with eight glaucoma cases (3.1%) being diagnosed after VP shunt operation. Among the types of glaucoma observed in the full patient cohort, there were nine cases of POAG, 12 cases of PEXG, one case of NTG, one case of PACG, one case of secondary glaucoma, and five cases of unspecified glaucoma. This distribution accounted for 33.3%, 40.0%, 3.3%, 3.3%, 3.3%, and 16.7% of the total glaucoma cases, respectively.

## Discussion

Our study resembles trends in glaucoma prevalence and age-related increases among both shunted iNPH patients and non-iNPH shunted patients when compared with population-based studies estimating glaucoma prevalence in Finland [14]. Presumed glaucoma prevalence was slightly higher in both older age groups, however our data does not support findings of markedly elevated prevalence of glaucoma among shunted iNPH patients compared to the prevalence of age-matched controls in Finland. The difference in glaucoma prevalence could also be partly attributed to the comprehensive individual examination of the study group patients, as opposed to the methods used in a population-based study. Out of the total 262 patients, there were 30 cases of glaucoma, including 8 cases diagnosed after the shunt operation. It is important to note that the time between shunt acquisitions varied significantly within the cohort, ranging from the earliest patients receiving shunts in January 2009 to the most recent in June 2023. This variation could potentially influence the results when assessing the impact of shunting on glaucoma prevalence. Despite this variability, it could be argued that the effect of shunting does not appear to correlate with a markedly increased risk of glaucoma. A prospective study by Gallina et al. [20], found increased rates of NTG in NPH patients who were treated with VP shunt, suggesting a potential link between intracranial pressure and NTG development. The causes of disparities in the results are potential differences in methods, study population and the number of participants (Gallina et al. *n* = 22). Our findings are further supported by our earlier study in which a cohort of shunted iNPH patients from the Kuopio iNPH registry underwent ophthalmological examinations, revealing no increased rate of glaucoma [26].

The limited number of glaucoma cases hinders the comparative analysis of subtypes, especially with the marked percentage of unspecified glaucoma cases. In the earlier data on glaucoma subtypes in Finland, the three most common types were primary POAG constituting approximately 36.6–44.9% of all cases, followed by PEXG at 20.3–29.3%, and NTG at 5.1–9.7% of all cases [27]. A similar trend was observed in the NPH (+) group for POAG and PEXG, but without any cases of NTG.

Previous studies have indicated a higher prevalence of hypertension among iNPH patients as opposed to matched controls diagnosed with other neurological or neurosurgical conditions [28]. In our comparison between iNPH (+) and iNPH (-) groups, hypertension did not significantly differ between the groups. Analysis from extensive population studies illustrates age-specific trends in hypertension prevalence among men and women. In Finland, the prevalence of hypertension among women increases with age, from 66% in the 60–69 age group to 82% among those aged 70–79. Similarly, men within these age brackets display rates of 70% for ages 60–69 and 76% for the 70–79 age group [29]. Our study groups do not seem to demonstrate an elevated age-adjusted hypertension prevalence.

The combined prevalence of T2DM and T1DM in Finland is approximately 8%, with 400,000 cases of type 2 diabetes and 50,000 of type 1 diabetes [30]. Data on age-stratified diabetes prevalences in Finland is limited. In a study focusing on the elderly Finnish population aged 70 and above, the total prevalence of diabetes was recorded at 22.0% [31]. However, an older study focusing on diabetes prevalence among men aged 65 to 84 years, particularly in the eastern region of Finland, indicated notable findings when stratified by age groups. In this study, age-specific prevalence rates were observed within age categories of 65–69, 70–74, 75–79, and 80–85, revealing prevalence rates of 22.5%, 29.0%, 45.5%, and 25.0%, respectively [32]. When considering previous studies and the mean age (74.6 years) of the NPH (+) group at the point of obtaining comorbidity data, the prevalence of diabetes may be higher compared to the general prevalence in age-matched populations in Finland. Elevated diabetes prevalence has been observed among familial NPH patients in contrast to their non-iNPH relatives [33] as well as within the iNPH population compared to age- and cohort-matched non-iNPH controls [34]. In our study, we did not find a statistically significant difference between the groups. In part, this observation can be attributed to the fact that the comparison group comprises many individuals with various neurodegenerative conditions rather than a neurologically healthy control group. Some of these conditions are recognized for their association with T2DM [35]. This supports the notion of earlier studies, suggesting that T2DM could be a possible risk factor for iNPH and may contribute to its pathogenesis.

The role of Aβ and HPτ in glaucoma remains unclear. Ongoing research aims to uncover potential shared pathogenic mechanisms between retinal and brain degeneration. It has been hypothesized that Aβ and HPτ could contribute to glaucomatous degeneration. Aβ and HPτ have been extensively studied, especially in Alzheimer’s disease, where their deposits together can cause neuroinflammation and disrupt brain iron homeostasis, leading to progressive neuronal death and dementia. Similarly, in the eye, inflammation mediated by the accumulation of Aβ and HPτ in retinal ganglion cells suggests overlapping pathology [36]. Our study cohort included patients with various neurological conditions often associated with Aβ and HPτ. Both the iNPH (+) and iNPH (-) groups displayed similar Aβ and HPτ biopsy profiles, and the prevalence of glaucoma resembled that of their peers.

This study features a distinctive cohort of extensively examined shunted iNPH patients. Given the scarcity of glaucoma data in iNPH populations and with often limited sample sizes in the existing studies, our research partially aims to address this gap. To ensure the reliability of our findings, we individually mined patient data from multiple sources to obtain up-to-date information on glaucoma status. This study has its limitations, including variability in shunt acquisition time, which may introduce confounding factors when assessing the impact of shunting on glaucoma prevalence. Known glaucoma risk factors could be only partially included in the study with the data available. However, based on the material we had, it seems that optic nerve morphological changes or cardiovascular risks, such as hypertension are not associated with iNPH development. Additionally, potential selection bias should be noted due to the exclusion of patients residing outside of the KUH area and the cut-off based on system reliability. The study lacks a healthy comparison group, with the iNPH (-) group consisting of patients suffering from various neurodegenerative and other neurological condition which could potentially affect the comparison results.

In conclusion, our findings suggest that shunted iNPH patients do not display a markedly elevated prevalence of glaucoma compared to their age-matched peers or patients with other neurodegenerative and neurological conditions contrary to previous studies on iNPH populations. This disparity in results could be attributed to differences in population, sample size, methodologies, and limitations across various studies.

## Data Availability

The data supporting the results of this study can be obtained upon request from the corresponding author. These data are not accessible to the public due to privacy and ethical constraints.
